# Transcriptomic and proteomic profiling of peptidase expression in *Fasciola hepatica* eggs developing at host’s body temperature

**DOI:** 10.1038/s41598-022-14419-z

**Published:** 2022-06-20

**Authors:** Jana Ilgová, Jiří Vorel, Pavel Roudnický, Lucie Škorpíková, Martin Horn, Martin Kašný

**Affiliations:** 1 Department of Botany and Zoology, Faculty of Science, Masaryk University, 611 37 Brno, Czech Republic; 2 Central European Institute of Technology, Masaryk University, 625 00 Brno, Czech Republic; 3 Institute of Organic Chemistry and Biochemistry, Czech Academy of Sciences, 166 10 Prague, Czech Republic

**Keywords:** Biochemistry, Computational biology and bioinformatics, Zoology

## Abstract

*Fasciola hepatica* is a global parasite of livestock which also causes a neglected zoonosis in humans. The parasite’s communication with the host during its complicated lifecycle is based on an ingenious enzymatic apparatus which includes a variety of peptidases. These enzymes are implicated in parasite migration, pathogenesis of the disease, and modification of host immune response. Although the dynamics of proteolytic machinery produced by intra-mammalian *F. hepatica* life stages has been previously investigated in great detail, peptidases of the eggs so far received little scientific attention. In this study, we performed a comparative RNA-seq analysis aimed at identification of peptidases expressed in *F. hepatica* eggs, cultured at 37 °C to represent gall bladder retained eggs, for different time periods and employed mass spectrometry in order to identify and quantify peptidases translated in *F. hepatica* egg lysates. We demonstrated that *F. hepatica* eggs undergo significant molecular changes when cultured at the physiological temperature of the definitive host. Egg transcriptome is subject to numerous subtle changes while their proteome is even more variable. The peptidase profile is considerably modified on both transcriptome and proteome level. Finally, we measured and classified proteolytic activities in extracts from *F. hepatica* eggs using a library of fluorogenic substrates and peptidase class-selective inhibitors. Activities of threonine peptidases were detected constantly, while the cysteine peptidases prevailing in freshly laid eggs are substituted by aspartic peptidase and metallopeptidase activities in the later stages of egg development.

## Introduction

The liver fluke *Fasciola hepatica* (Trematoda, Fasciolidae) causes a neglected foodborne zoonosis known as fascioliasis, which afflicts millions of people and livestock all over the world^1^. Besides infecting about 50 million people worldwide^2^, this parasite also contributes to significant economic losses in livestock production (estimated at approx. US$ 3.2 billion a year^3^). The clinical picture of fascioliasis, in both humans and livestock, includes anaemia, bile duct fibrosis and inflammation, liver atrophy, cirrhosis, decrease in bile production, and weight loss^4–7^.

This parasite’s complex lifecycle begins with infection of an intermediate snail host (family Lymnaeidae) by freshly hatched miracidia. After clonal asexual reproduction, the newly formed larval stages (cercariae) exit the snail and form cysts (metacercariae) on aquatic vegetation. Upon ingestion of the cyst by the definitive mammalian host, the newly excysted juveniles (NEJ) migrate to the host’s liver parenchyma, where they cause severe damage. Maturation of *F. hepatica* individuals occurs in the bile ducts while the infection progresses to a chronic stage. Up to 50,000 eggs a day are released via faeces to the environment^8^, where the development of miracidia begins again. In the presence of water, stimulation by light changes the permeability of internal eggshell surface, which results in the hatching of miracidium^9^.

Drug resistance against triclabendazole, which started to quickly spread in recent decades^10^, led to an intensification of research of this species using genomics, transcriptomics, and proteomics^11^. Publication of its genome^12,13^, stage-specific transcriptomes^12–19^, somatic proteomes^14,16,20,21^, secretomes^14,22–25^, and glycoproteomes^26,27^ then shed light on the general biology of *F. hepatica*, specific features of its life stages, as well as its interaction with the host, including manipulation of host immune system or pathogenesis.

Although eggs are crucial for the spread of this parasite and their embryonic development is well documented on a microscopic level^28,29^, few studies so far employed a bioinformatic approach to the investigation of biology of this life stage. Moxon et al.^21^ refer to mass-spectrometric identification of proteins separated by two-dimensional electrophoresis, which are expressed by the embryonic stage of *F. hepatica* cultivated in 30 °C. In their study, they observed mainly antioxidant proteins and protein chaperones and described some changes in protein composition during embryonation. Information about the amount of time required for the passage of eggs from the bile ducts to faeces is scarce. It is possible that eggs of *F. hepatica* undergo changes during their journey to the outer environment and release proteins which might interact with the molecules of the host, but studies focused on molecular changes in eggs that occur in the host are missing.

Peptidases, enzymes which became highly differentiated among various trematodes^13^, mediate numerous defence and homeostatic processes including protein metabolism, folding, activation or degradation, tissue remodelling, intracellular signalling, migration, and evasion of host immune response^30–32^. Previous research of related species from genus *Schistosoma* had shown that most proteins secreted by schistosome eggs are involved in catalytic processes including proteolysis^33^. Exceptional in this aspect are cysteine peptidases (cathepsin L, cathepsin B, and legumain), which had undergone a remarkable sequential and functional diversification^11^.

In an effort to further our understanding of *F. hepatica* egg biology, we performed a comparative RNA-sequencing and mass spectrometry analyses of transcriptome and proteome changes in *F. hepatica* eggs at three time points (0, 5, and 10 days post laying) during development with focus on peptidases. Our aim was to reveal the range of peptidase genes transcribed and translated by eggs during their passage through the host’s liver, gallbladder, and gut to the outer environment.

## Material and methods

### Collection of *Fasciola hepatica* eggs

Adult individuals of *F. hepatica* were collected from the bile ducts of naturally infected bovine livers obtained in cooperation with the local abattoir from a farm in the South Bohemian Region (Czech Republic). The worms were washed three times in 10 mM PBS buffer (pH 7.8) with 150 mM NaCl and then transferred to RPMI 1640 medium (tempered to 37 °C) containing glucose (55 mM), HEPES (15 mM), penicillin (50 U ml^−1^), and streptomycin (50 µg ml^−1^). After a 4-h incubation at 37 °C, the medium with freshly laid eggs was collected and exchanged for a fresh one. This process was repeated twice more for a total of 12 h of incubation. Harvested eggs were counted using a Bürker chamber and evenly divided into three groups (T0, T5, T10), each with three separate replicates. Eggs from the first group (T0) were washed in sterilised water and frozen immediately at − 80 °C. Eggs from groups T5 and T10 were incubated in the dark in sterilised water supplemented with penicillin (50 U ml^−1^) and streptomycin (50 µg ml^−1^) for five (group T5) and 10 days (group T10) at 37 °C, after which the eggs were collected and frozen at − 80 °C until further use.

### Viability assay

In order to test whether incubation at 37 °C affects the egg viability, an independent hatching experiment was performed. Freshly laid eggs were distributed into 24-well cultivation plates (100 eggs per well) in sterilised water supplemented with penicillin (50 U ml^−1^) and streptomycin (50 µg ml^−1^). Three experimental groups, control, T5, and T10 were used, each in quintuplicates. Eggs from control group were cultivated at 25 °C in dark throughout the whole experiment as described in Fairweather et al.^34^. Eggs from T5 and T10 groups were cultivated in a dark incubator at 37 °C for 5 and 10 days, respectively. After this period the cultivation temperature was decreased to 25 °C, thereby simulating the release of eggs from the host body to the external environment. All plates were regularly checked under the microscope. At day 14 post laying, plates with developing eggs were exposed to light for 2 h and eggs at each stage of development (cell division stage, eye-spot stage, dead, hatched) were counted under inverted microscope and the micrographs were taken using Olympus BX51 microscope. Plates were returned to the incubator and the light stimulated hatching procedure was repeated on days 16, 19, 23, 26, 30, 35 post laying, until no new hatched eggs and/or swimming miracidia were observed. Graph showing the hatch rate in each group was generated using GraphPad Prism 8.

### RNA isolation, library preparation and sequencing

TRIzol Reagent (Invitrogen) was added to each sample containing approx. 7500 *F. hepatica* eggs. Eggs were mechanically homogenised and total RNA extracted using the phenol–chloroform method according to TRIzol manufacturer’s instructions. DNases were inactivated using a DNA-free DNA Removal Kit (Invitrogen). Concentration and purity of the extracted RNA were measured using NanoDrop 8000 Spectrophotometer (Thermo Fisher Scientific). Subsequent steps, library preparation, and sequencing were carried out by the CEITEC Genomics Core Facility (Masaryk University Brno, Czech Republic). A total of nine libraries were prepared by strand-specific QuantSeq 3′ mRNA-Seq FWD kit (Lexogen) according to the manufacturer’s guidelines. From total RNA input, the first strands of cDNA molecules were amplified by oligo(dT) primers containing the Illumina-specific Read 2 linker sequence. After the degradation of RNA templates, second strands were synthesised by random priming with the Illumina-specific Read 1 linker sequence. The obtained double-stranded libraries were purified by magnetic beads. Finally, cDNA libraries were amplified with specific adapters required for cluster generation and purified from PCR components on magnetic beads. Prepared libraries were sequenced in one pool on Illumina NextSeq 500 platform (single-end sequencing, 70 bp-long reads).

### Preparation of *Fasciola hepatica* egg lysates for LC–MS/MS analyses

For mass spectrometric analyses, we used approx. 600 eggs per replicate in each group. *F. hepatica* eggs were lysed in SDT buffer (4% SDS, 0.1 M DTT, 0.1 M Tris/HCl, pH 7.6) in a thermomixer (Eppendorf ThermoMixer C, 30 min, 95 °C, 750 rpm) with added glass beads. After that, the samples were centrifuged (15 min, 20,000×*g*) and collected supernatants used for filter-aided sample preparation (FASP) as described elsewhere^35^ using 0.5 μg of trypsin per sample (sequencing grade, Promega Corporation). The resulting peptides were used for LC–MS/MS analyses. Once sample preparation was complete, the total amount of peptides was estimated using RSLCnano system online coupled with HCTUltra ion trap (Bruker Corporation), which is based on the area under total ion current curve using MEC cell line tryptic digest as an external calibrant.

### Reannotation and identification of peptidases from the reference proteome

Proteins derived from the reference *F. hepatica* genome (PRJEB25283) deposited in WormBaseParasite database^36^ were reannotated by searching for the closest sequential homologues in the UniProt/UniRef100 protein database^37^ and in MEROPS database of peptidases^38^ using Diamond v0.9.34 (BLASTp algorithm, E-value cut-off 1e^−5^)^39^. Both databases were used in versions updated to June 2020. To prevent misleading annotations given by the MEROPS database, homologues to the *F. hepatica* proteins described as *unassigned peptidases*, *non-peptidase homologues*, or *hypothetical proteins* were additionally removed from the annotation as well as hits with E-value scores above 1e^−40^. GO terms and functional analyses were performed by InterProScan v5.44-79.0^40^.

### Sequencing, processing of raw reads, and quantification of transcripts

The quality of short single-end Illumina raw reads was visualised and evaluated using FastQC v0.11^41^. Adapter sequences and low-quality bases were trimmed using Trimmomatic v0.39^42^ and the trimmed reads were used for quantification of protein-coding transcripts derived from the reference *F. hepatica* genome by RSEM v1.3.3 using the Bowtie2 v2.3.0 alignment program^43^.

### LC–MS/MS analyses and data evaluation

LC–MS/MS analyses of all peptide mixtures were performed using UltiMate 3000 RSLCnano system connected to Orbitrap Fusion Lumos Tribrid spectrometer (Thermo Fisher Scientific). Prior to LC separation, tryptic digests were online concentrated and desalted using a trapping column (X-Bridge BEH 130 C18, dimensions 30 mm × 100 µm, 3.5 μm particles; Waters). After washing the trapping column with 0.1% formic acid (FA), the peptides were eluted in backflush mode (flow 0.3 µl min^−1^) from the trapping column onto an analytical column (Acclaim Pepmap100 C18, 3 µm particles, 75 μm × 500 mm; Thermo Fisher Scientific) during a 130 min gradient (1–80% of mobile phase B; mobile phase A: 0.1% FA in water; mobile phase B: 0.1% FA in 80% ACN). MS data were acquired in a data-dependent mode, selecting up to 20 precursors based on precursor abundance in a survey scan. Resolution of the survey scan was 120,000 (350–2000 m/z) with a target value of 4 × 10^5^ ions and maximum injection time of 100 ms. The MS/MS spectra were acquired with a target value of 5 × 10^4^ ions (resolution 15,000 at 110 m/z) and maximum injection time of 22 ms. The isolation window for fragmentation was set to 1.2 m/z. For data evaluation, we used MaxQuant software v1.6.10.43^44^. We conducted searches against the following inhouse-made protein databases: *F. hepatica* 9708 proteins derived from reference genome PRJEB25283 (updated to December 2019), *Bos taurus* 23,846 proteins from the UniProt reference proteome UP000009136 (presumed source of contamination), and cRAP contaminants^45^. Modifications for all database searches were set as follows: oxidation (M) and deamidation (N, Q) as variable modifications, and carbamidomethylation (C) as a fixed modification. Enzyme specificity was tryptic with two permissible miscleavages. Only peptides and proteins with false discovery rate threshold under 0.01 and proteins with at least two peptide identifications in at least one replicate were considered. For the purpose of this article, protein groups reported by MaxQuant are referred to as proteins. Intensities of reported proteins were further evaluated using the KNIME_docker_vnc v3.7.1a software^46^. Processing workflow is available upon request: it covers decoy hits and removal of contaminant protein groups (cRAP), protein group intensities, log2 transformation, and normalisation (loessF).

### Analyses of gene expression

To identify the differentially expressed (DE) genes on a transcript level, we used the obtained non-normalised *expected count* values for a DE analysis performed by DESeq2 v1.29.6^47^ in R v4.0.1^48^. We analysed DE transcripts in pairs of samples representing each time point (T0 vs T5, T0 vs T10, and T5 vs T10). Transcripts were considered significantly differentially expressed only if were expressed in both compared samples with log2fc > 1 or <  − 1 with adjusted P-value cut-off point of 0.05. In case of LC–MS/MS, comparative analyses between all samples were carried out using Limma v3.6.3^49^ based on the adjusted P-value cut-off of 0.05. Heatmaps reflecting gene/protein expression differences were generated in GraphPad Prism 8 using relative abundance values after logarithmic and Z-score transformation.

### Identification of differentially expanded cysteine peptidases

A detailed expression analysis was performed on selected isoforms of cysteine peptidases, cathepsin L, cathepsin B, and legumain, which differentially expand in the trematodes. Sequences derived from the *F. hepatica* genome assembly (PRJEB25283) were curated manually using sequences described in McNulty et al.^13^ and Cwiklinski et al.^12,50^. Individual isoforms of cysteine peptidases identified in the present study were named according to the classification presented in Cwiklinski et al.^50^.

### Preparation of *Fasciola hepatica* egg protein extract for biochemical analysis

Soluble protein extracts (2 mg ml^−1^) were prepared from approx. 50,000 eggs collected at each time point (0, 5, and 10 days post laying) by mechanical homogenisation on ice in 0.1 M Tris/HCl, pH 7.3, containing 1% CHAPS, followed by sonication (Biologics 150 VT, 3.8 mm micro tip, 30% amplitude, 25% pulse, 15 min). The extracts were cleared by centrifugation (16,000×*g* at 4 °C for 45 min) and stored at − 80 °C before use. Protein concentration in the prepared extracts was determined using Quaint-iT™ Protein Assay Kit (Life Technologies).

### Preparation of substrates

Fluorescence resonance energy transfer (FRET) substrates containing (7-methoxycoumarin-4-yl)acetic acid (MCA) as the fluorescent group and 2,4-dinitro-phenyl lysine (Lys(Dnp)) as the quencher acceptor were synthesised as peptidyl amides by Fmoc solid-phase chemistry in an ABI 433A peptide synthesiser (Applied Biosystems), purified by reverse-phase (RP)-HPLC over a C18 column using the TFA/acetonitrile system and characterised by electrospray ionisation mass spectrometry on an LCQ Classic Finnigan Mat device (Thermo Finnigan) as described previously^51,52^ or purchased from Sigma, Bachem, or R&D systems (see Supplementary file 1).

### Peptidase activity and inhibition assays

Proteolytic activities in *F. hepatica* egg extracts were measured in a continuous kinetic assay using a mixture of internally quenched FRET substrates (1 µM each) (Supplementary file 1). We selected diverse substrate sequences to enable the detection of various protease classes. An analogous panel of fluorescent peptide substrates has been previously used to detect proteolytic activity in the excretory/secretory products of *Schistosoma mansoni* eggs^52^ or freshwater planaria *Schmidtea mediteranea*^53^. In our study, measurements were performed at 37 °C in 96-well plates in total volume of 100 µl using an aliquot of protein extract (20 µg ml^−1^) and the following assay buffers: 0.15 M citrate–phosphate buffer (pH 2.5–8) or 0.1 M sodium borate buffer (pH 7.5–10), both containing 100 mM NaCl, 0.05% PEG 1000, and 1.25 mM DTT. Proteolytic activity was continuously measured after addition of the substrate mixture in a SpectraMax i3 spectrofluorometer (Molecular Devices) at 325 nm excitation and 392 nm emission wavelengths. All assays were performed in triplicate. Data were analysed using GraphPad Prism 8 and activities expressed as relative fluorescent units hydrolysed per second per µg of protein in the extract (RFU s^−1^ µg^−1^). Data were normalised against the highest proteolytic activity recorded for each protein sample. For activity assay in the presence of peptidase inhibitors, the extracts were preincubated for 10 min with either 1 mM EDTA, 10 µM E-64, 1 mM AEBSF, 10 µM pepstatin, and 10 µM MG132, or their mixture. The remaining proteolytic activity was measured in pH 3.5, 6.0, and 8.5 after addition of the substrate under the same conditions as described above and expressed as a percentage of remaining activity relative to the uninhibited control.

### Ethics statement

All procedures performed in studies involving animals were carried out in accordance with European Directive 2010/63/EU and Czech laws 246/1992 and 359/2012 which regulate research involving animals.

## Results

From the bovine liver, we isolated 97 live *F. hepatica* adults. After overnight cultivation, we recovered approx. 228,000 laid eggs, which we divided in three groups. The first group (T0) was immediately frozen at − 80 °C, while the other two groups (T5 and T10) were incubated for 5 and 10 days at 37 °C, respectively. Before incubation and/or freezing, the eggs from each time point were subdivided according to the type of subsequent analysis as follows: 21,000 eggs for RNA sequencing (RNA-seq), 1800 eggs for mass spectrometry analysis, and 50,000 eggs for a biochemical analysis of proteolytic activity. Material for RNA-seq and LC–MS/MS analyses from each time point was divided in three replicates.

### Egg viability assay

**Figure 1:**
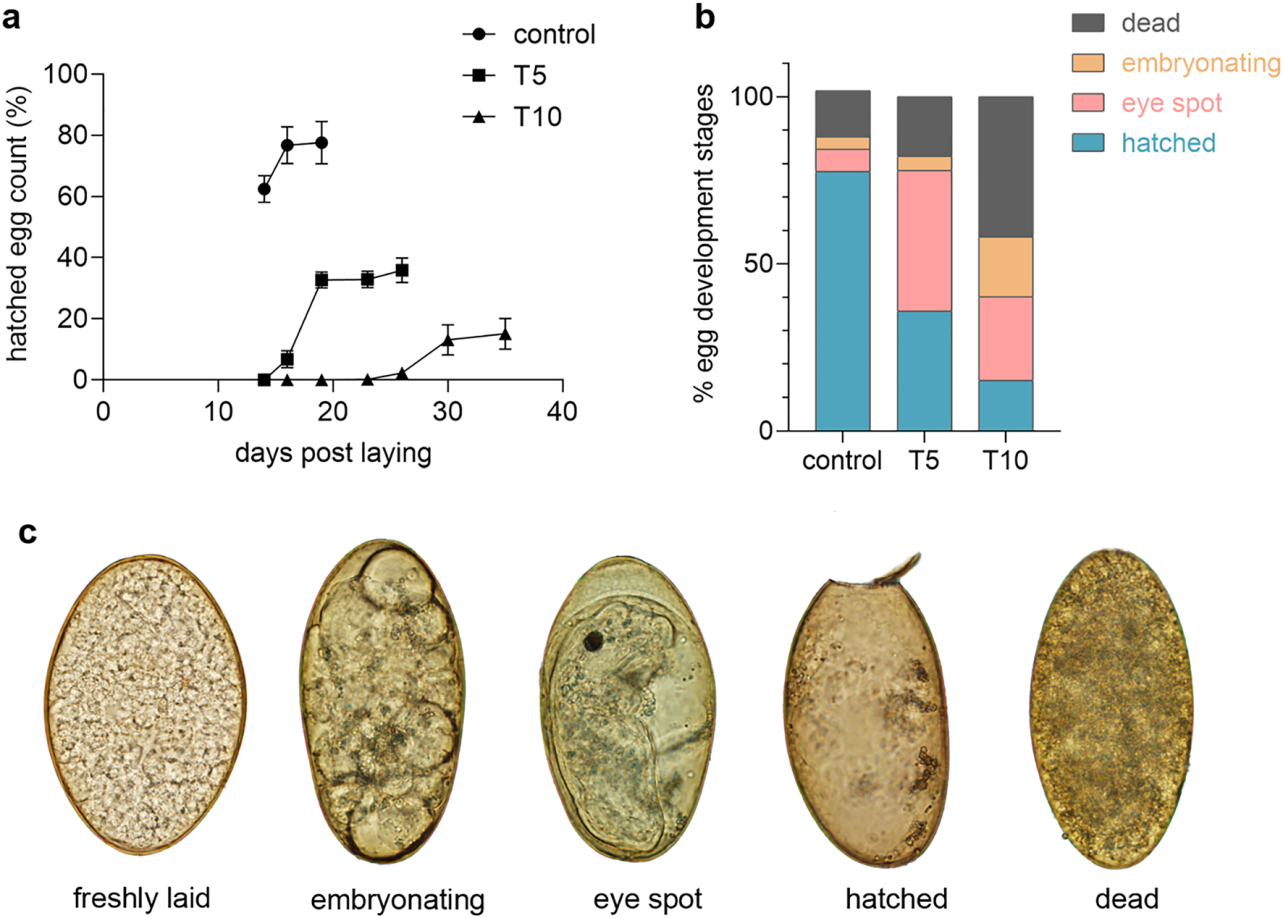
Egg viability assay. (**a**) Hatch rate dynamics during the egg development. Numbers of hatched eggs in groups the control, T5 and T10 group are presented as percentage of total number of eggs (mean and standard deviation are given). (**b**) Proportion of four egg development stages. Proportion of eggs from each stage on the day when last newly hatched eggs were observed in the particular group, that is on day 19, 26 and 35 in control, T5 and T10 groups, respectively. (**c**) Light micrographs illustrating the different stages of egg development. Freshly laid egg, embryonating egg at cell division stage, eye spot stage, hatched egg with an open operculum after the release of miracidium, dead egg (from left to right).

The effect of incubation at 37 °C on egg development and viability was evaluated using a light-stimulated egg hatch test. Freshly laid eggs were cultivated for 5 and 10 days at 37 °C (T5 and T10 groups, respectively), then the temperature was lowered, and egg developed at 25 °C. Eggs maintained at 25 °C served as a control group. The hatching tests were performed on day 14, 16, 19, 23, 26, 30 and 35 post laying. Eggs were checked regularly until a release of new miracidia was not recorded. In addition to the hatching tests, eggs were regularly inspected with light microscope (Fig. 1c) for visible signs of embryonation characterised by the cell division, which were observed on day 5 in all groups. However, hatching of eggs from groups incubated at 37 °C was substantially delayed comparing to the control group maintained at 25 °C throughout the whole experiment. Approximately 78% of eggs from control group embryonating all time at 25 °C successfully released miracidia at days 14, 16 and 19 (Fig. 1a). Releasing miracidia in group T5 begun on day 16 (7% of eggs hatched) and ended on day 26 with 36% hatched eggs in total. Even more pronounced retardation of development was observed in eggs from group T10, where the hatching started on day 26 (2% of eggs hatched) and ended on day 35 with only 15% of eggs hatched. The egg death rate increased with the length of incubation at 37 °C reaching up to 14, 18 and 42% of total amount of eggs in control, T5 and T10 group, respectively (Fig. 1b). Nevertheless, signs of partial or complete embryonation (eye spot stage, cell division stage, hatched egg) was reported in 82 and 58% of eggs in T5 and T10 groups, respectively.

### Sequencing, annotation, and peptidases identification

Single-end sequencing generated about 23 million short reads for each sample (Supplementary file 2). A total of 9708 *F. hepatica* proteins derived from the reference genome PRJEB25283^36^ were reannotated using UniProt/UniRef100 and MEROPS protein database; 8159 homologues were found in the UniProt database (Supplementary file 3). Moreover, the GO term analysis revealed 1879 unique GO terms across all *F. hepatica* reference proteins, whereby 73 GO terms were related to proteolytic activity (Supplementary file 4) and we used them to identify a total of 193 peptidases.

### Transcripts and proteins present at different time points of egg development

Protein-coding gene transcripts and translated proteins were considered transcribed/translated in a particular time-point group when the median of relative quantification values in TPM (transcripts per million) given by RNA-seq and PPM (parts per million) given by LC–MS/MS of all three replicates was non-zero (for all data, see Supplementary file 5).

**Figure 2:**
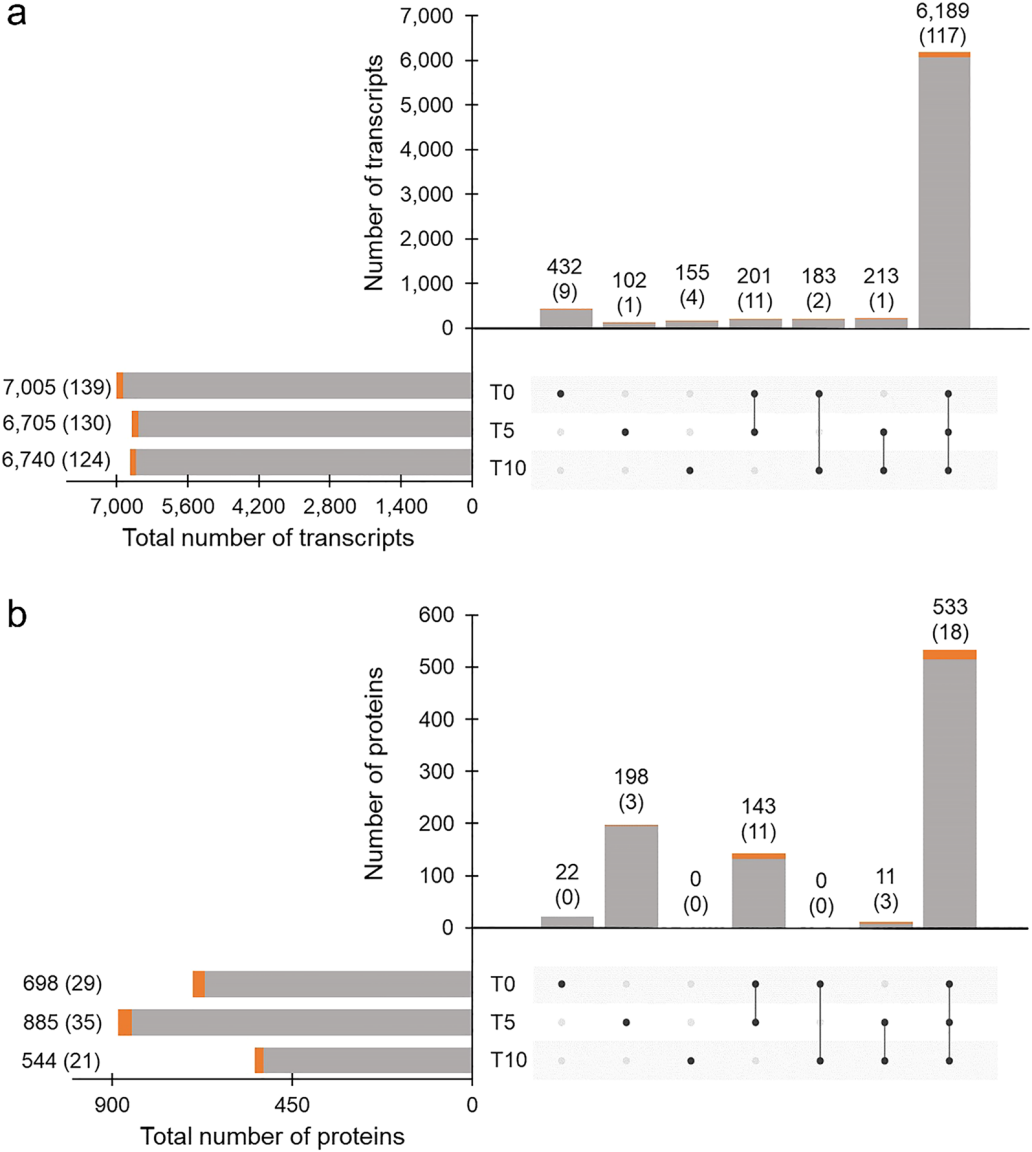
UpSet plots depicting the number of transcripts and proteins quantified in *F. hepatica* eggs at different points of development. The numbers represent a distribution of protein-coding gene transcripts (**a**) and translated proteins (**b**) at three different points of egg development (T0, T5, and T10). Solitary dots represent transcripts or proteins unique to each time point; two or three dots connected by a line represent the number of transcripts or proteins shared between two or three different time points, respectively. The numbers of quantified peptidases are given in parentheses and highlighted in orange.

Among the 9708 reference proteins, 7475 (77.0%) protein-coding transcripts of *F. hepatica* eggs were quantified by RNA-seq in at least one time point. Regarding a comparison between the time-point groups, the numbers of transcribed sequences identified in T0, T5, and T10 samples were 7005, 6705, and 6740, respectively (Fig. 2a). Transcription of a vast majority of identified transcripts (6189, that is approx. 83% of all identified transcripts) was detected in eggs throughout the monitored egg development, i.e., in all three time-point groups. The proportion of 6189 shared transcripts from the total transcripts quantified at time points T0, T5, T10 was 88%, 92%, and 91%, respectively.

About 200 transcripts were shared only between pairs of groups (201 transcripts in T0 vs T5, 183 transcripts in T0 vs T10 and 213 transcripts in T5 vs T10 comparisons). Interestingly, 432 protein-coding genes were transcribed only in T0, that is, during early egg formation. Out of all 7475 transcribed genes, we determined 145 transcripts coding proteins with proteolytic activity (based on GO term analysis): this accounts for approx. 1.9% of all expressed transcripts. Majority of identified peptidase transcripts (117) were shared by eggs at all three time points (T0, T5, T10), similarly as for all detected protein-coding gene transcripts.

LC–MS/MS analyses revealed 907 translated proteins in *F. hepatica* eggs (9.3% of the 9708 reference proteins). As in the case of transcripts, the majority of produced proteins (533, approx. 59% of all identified proteins) was shared across the time points (Fig. 2b), but the proportion of shared proteins from all proteins detected at each point was 76% (T0), 60% (T5) and 98% (T10), which indicates that the proteome is more variable than the transcriptome. The data show that the highest level of unique protein translation tends to occur up to the fifth day of egg development. The number of proteins identified in the T10 group was considerably lower than in samples from the earlier time points. The most abundant proteins in all studied samples were antioxidant and detoxification enzymes (thioredoxin peroxidase, glutathione S-transferase), an iron-storage protein (ferritin), structural proteins (actin, tubulin, myoglobin), proteins important for energetic metabolism (glyceraldehyde-3-phosphate dehydrogenase) and heat-shock proteins (alpha-crystallin domain containing heat-shock protein). Approximately 3.9% (35 in total) of all quantified proteins were characterised as peptidases based on GO term analysis.

**Figure 3:**
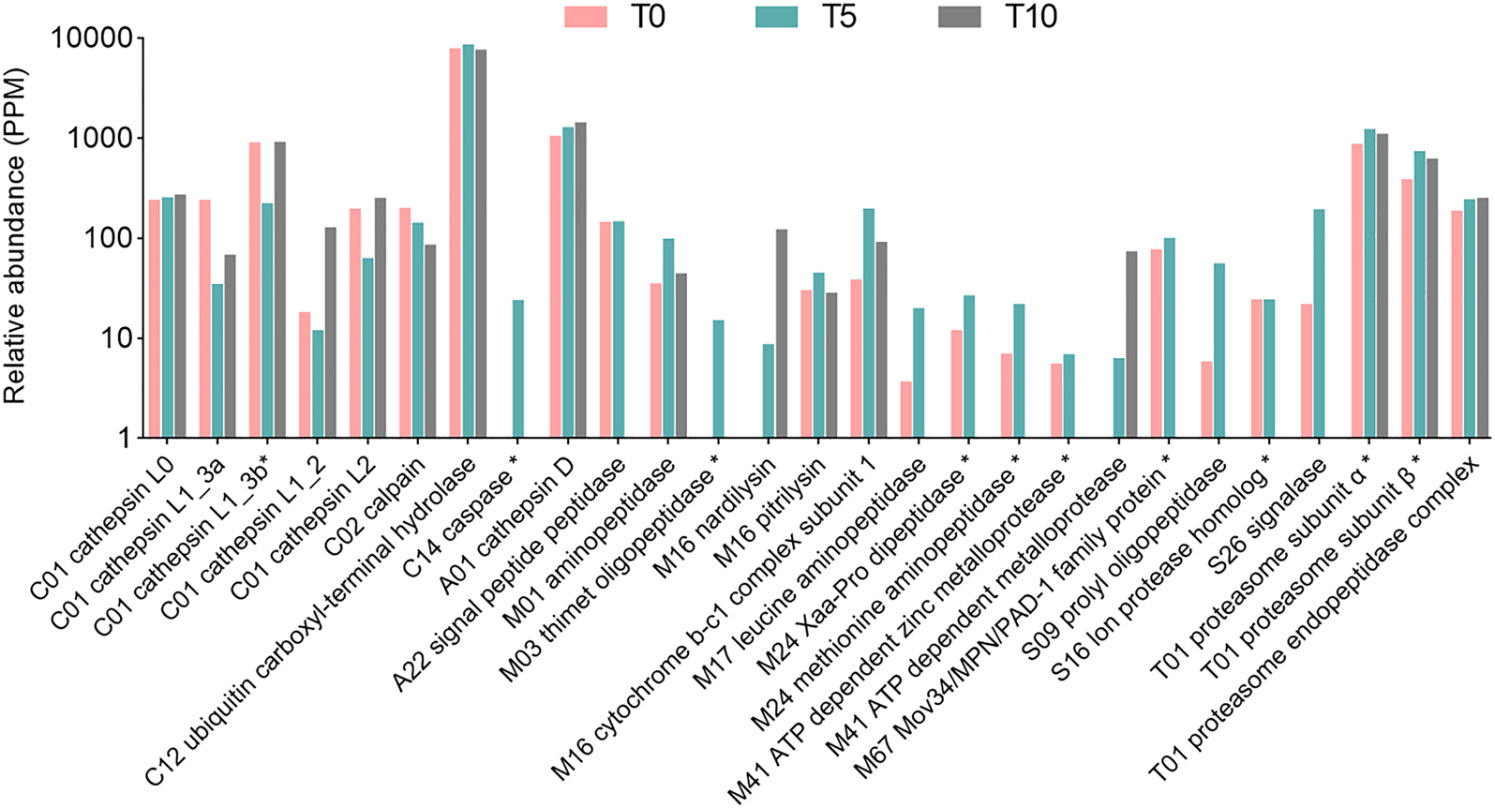
Relative abundances of peptidases identified by LC–MS/MS. Relative abundance values (PPM) are shown on the logarithmic scale. Different isoforms of signalase, proteasome subunit α, proteasome subunit β, and proteasome endopeptidase complex are presented as single columns. Peptidases marked by an asterisk (n = 10) were significantly differentially expressed. Cathepsin L isoforms were named according to Cwiklinski et al.^50^ nomenclature.

**Figure 4:**
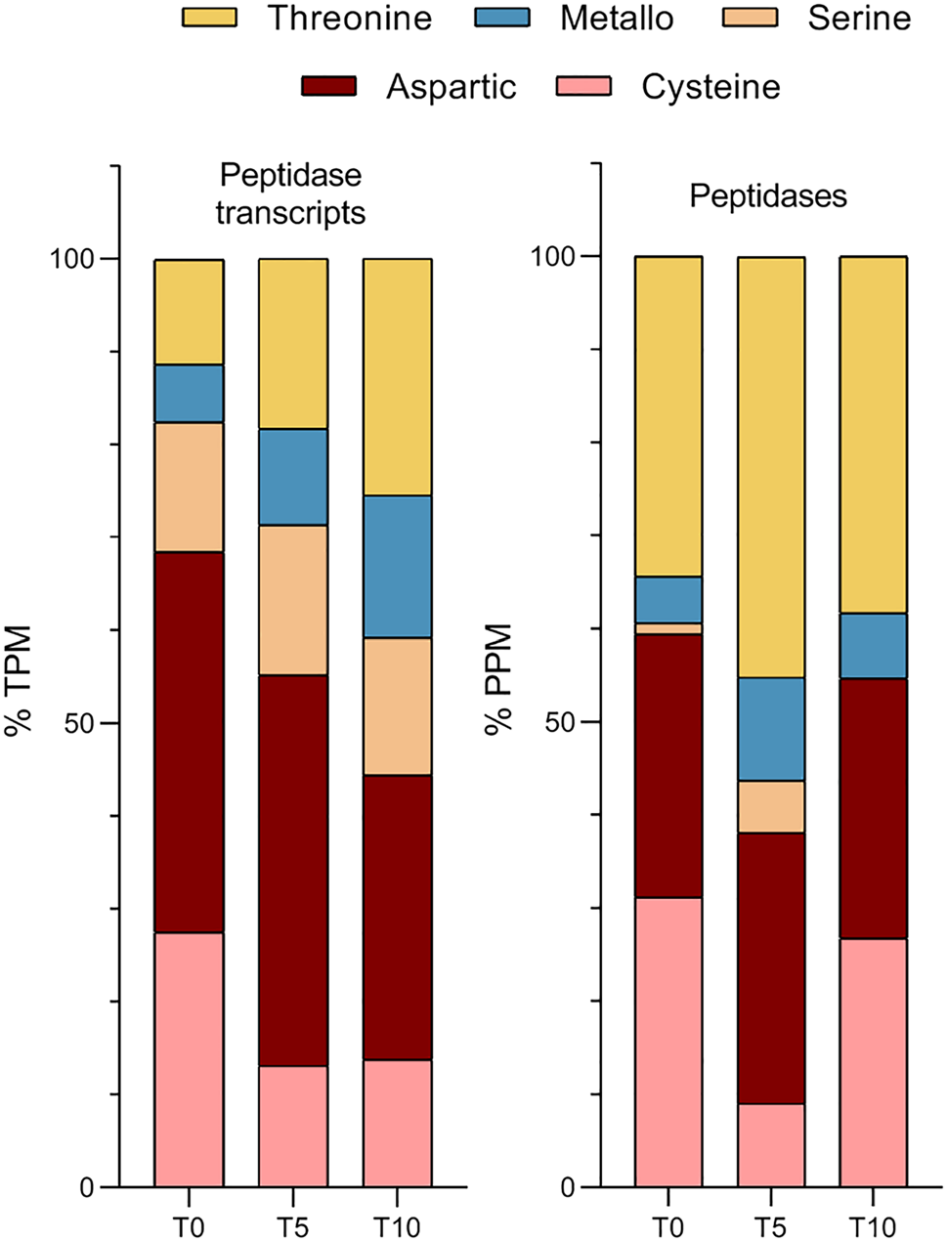
Representation of individual catalytic classes among the transcribed/translated peptidases. Percentages were counted based on relative abundance values in TPM/PPM.

Peptidase-coding transcripts and translated peptidases evaluated as the most abundant (based on the TPM/PPM values) in T0, T5, and T10 samples are listed in Table 1. Relative abundances of all proteins with assigned proteolytic activity identified by LC–MS/MS (n = 27) are shown in Fig. 3. The dynamics of peptidase class composition as evidenced by RNA-seq and MS data is not tightly correlated (Fig. 4). A large proportion of peptidase transcripts is comprised of aspartic peptidases, followed by cysteine and threonine peptidases. Finally, the lower expression of metallopeptidases is comparable with that of serine peptidases. On the protein level, threonine peptidases, which form a majority of translated peptidases, are followed by cysteine and aspartic peptidases, which represent about one quarter of the whole peptidase spectrum. The proportion of metallopeptidases and serine peptidases was, according to recorded PPM values, the lowest.

**Table 1:** Top ten most abundant peptidases identified in *F. hepatica* eggs at different points of development.

	Transcriptomics data				Proteomics data			
	#	Peptidase ID	Family	TPM	#	Peptidase	Family	PPM
T0	33	**Cathepsin D**	A01	3846.41	22	**Ubiquitin carboxyl terminal hydrolase**	C12	7866.39
	121	**Cathepsin L (*CL0*)**	C01	1157.89	135	**Cathepsin D**	A01	1047.99
	332	**Signalase subunit**	S26	413.73	156	Cathepsin L (***CL1_3b***)	C01	900.96
	463	Furin	S08	265.74	261	**Proteasome subunit α_2**	T01	378.09
	465	**Ubiquitin carboxyl terminal hydrolase**	C12	265.14	293	**Proteasome subunit α_1**	T01	290.42
	473	Signalase 2A	A22	255.07	314	**Secreted cathepsin L1 (*CL1_3a*)**	C01	242.53
	513	Cercarial peptidase	S01	227.85	316	**Cathepsin L (*CL0*)**	C01	240.05
	537	Peptidase S1 domain containing protein	S01	213.41	344	**Calpain-2 catalytic subunit**	C02	201.18
	553	Cathepsin B (***CB9***)	C01	202.27	345	**Secreted cathepsin L2 (*CL2*)**	C01	198.09
	578	**Secreted cathepsin L (*CL1_3a*)**	C01	191.84	353	**Proteasome subunit β_1**	T01	190.49
T5	33	**Cathepsin D**	A01	3387.20	18	**Ubiquitin carboxyl terminal hydrolase**	C12	8634.68
	161	**Cathepsin L (*CL0*)**	C01	963.85	125	**Cathepsin D**	A01	1288.93
	267	Cathepsin B (***CB9***)	C01	585.06	254	**Proteasome subunit α_2**	T01	393.11
	400	**Signalase subunit**	S26	370.72	291	**Proteasome subunit α_3**	T01	306.41
	486	**Proteasome subunit β_1**	T01	282.88	314	**Proteasome prosome macropain subunit β**	T01	262.86
	539	**Ubiquitin carboxyl terminal hydrolase**	C12	247.31	321	**Cathepsin L (*CL0*)**	C01	256.38
	552	**Mov34/MPN/PAD-1 family protein**	M67	239.47	331	**Proteasome subunit α_4**	T01	230.80
	567	Furin_1	S08	232.23	337	Cathepsin L (***CL1_3b***)	C01	223.67
	597	**Proteasome subunit α_1**	T01	218.11	342	**Proteasome subunit β_1**	T01	216.57
	613	Putative signalase	A22	209.55	361	**Cytochrome b-c1 subunit 1**	M16	197.33
T10	62	**Cathepsin D**	A01	2406.63	24	**Ubiquitin carboxyl terminal hydrolase**	C12	7602.25
	232	**Cathepsin L (*CL0*)**	C01	712.57	91	**Cathepsin D**	A01	1441.69
	371	Signalase subunit	S26	449.44	139	Cathepsin L (***CL1_3b***)	C01	916.26
	378	Cathepsin B (***CB9***)	C01	438.60	212	**Proteasome subunit α_1**	T01	468.51
	456	**Proteasome subunit β_1**	T01	349.47	223	**Proteasome subunit α_2**	T01	416.09
	464	Mov34/MPN/PAD-1 family protein	M67	342.65	260	**Proteasome prosome macropain subunit β**	T01	289.92
	531	Proteasome subunit β_2	T01	290.20	269	**Cathepsin L (*CL0*)**	C01	271.49
	569	**Proteasome subunit β_3**	T01	271.71	282	**Secreted cathepsin L2 (*CL2*)**	C01	252.02
	579	**Proteasome subunit α_1**	T01	266.31	286	**Proteasome subunit β_1**	T01	247.40
	690	Proteasome subunit β_4	T01	216.17	302	**Proteasome subunit α_4**	T01	220.22

The ranking of peptidases relative to the abundance of all quantified proteins is shown in column #. Peptidases quantified by both methods (RNA-seq and LC–MS/MS) at a given time point are highlighted in bold. Cathepsin L0, L1, and cathepsin B9 isoforms (bold italics) were named according to Cwiklinski et al.^50^ nomenclature.

### A comparison of transcriptome and proteome changes during egg development

Statistically significant changes in the gene expression were evaluated by comparing expression levels between pairs of samples of *F. hepatica* eggs; in the following, we refer to them as log_2_ fold change (log_2_FC). Data from an earlier development stage (T0 or T5) served as reference for analysing data from eggs belonging to a later stage of development (T5 or T10, respectively). All statistically significant changes in the expression (log_2_FC) are listed in Supplementary file 5. A comparative analysis of transcriptomic data resulted in the identification of 3577 unique transcripts significantly changed in at least one compared sample pair. Using > 1 and <  − 1 as thresholds of log_2_FC, the numbers of transcripts detected as significantly upregulated were 590, 927, and 107, whereas the numbers significantly downregulated were 287, 1112, and 85, when comparing the T0 vs. T5, T0 vs. T10, and T5 vs. T10 group, respectively. The number of proteins with significantly changed levels was notably lower: all in all, in the corresponding stage comparisons we detected 35, 0, and 44 upregulated and 40, 18 and 147 downregulated proteins. This suggests that the lowest number of changed transcripts—and conversely the highest number of changed proteins—was observed in a comparison of T5 vs. T10 time points.

**Figure 5:**
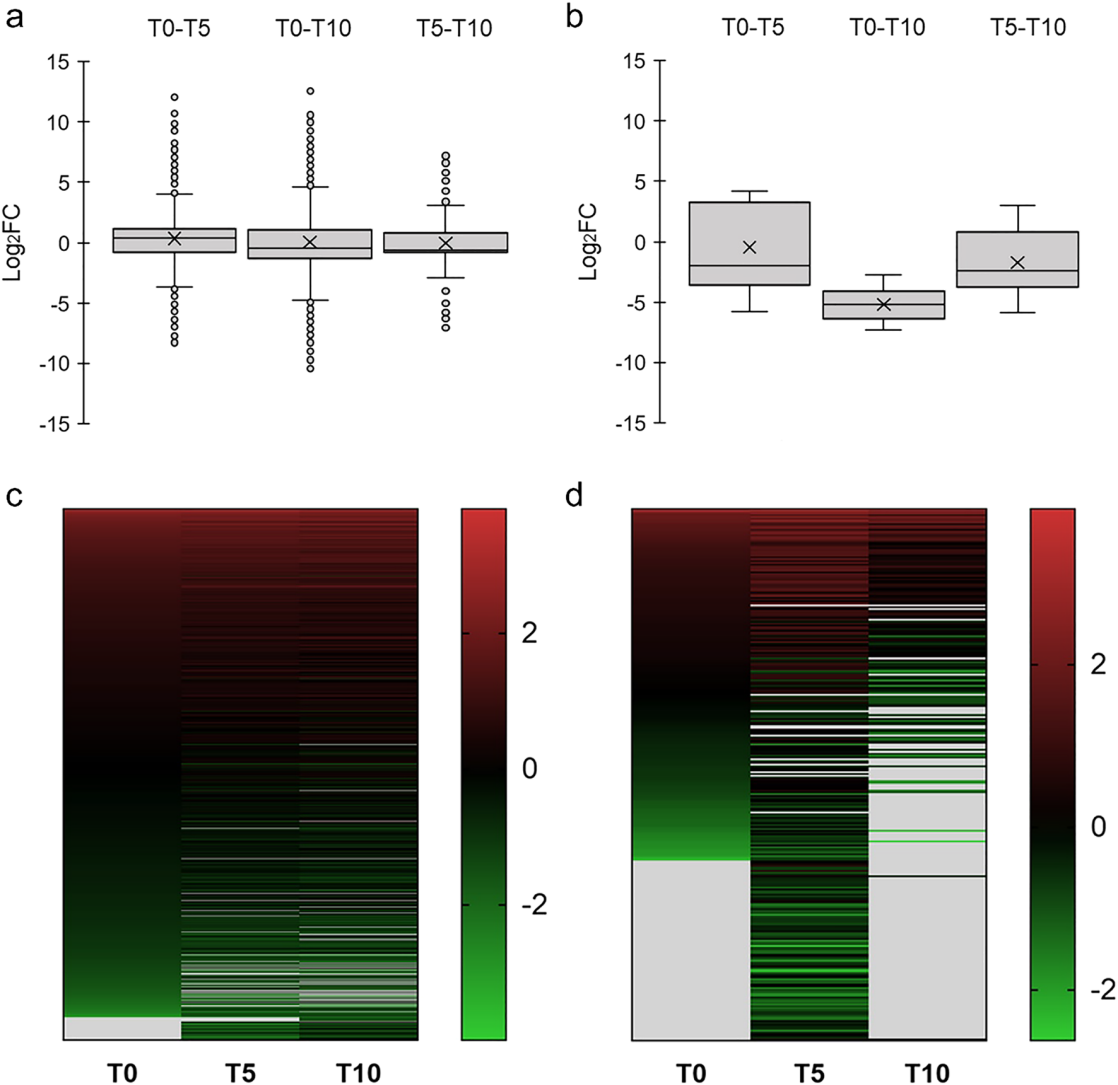
Statistically significant changes in the transcriptome and proteome in *F. hepatica* eggs at different points of development. The box plots provide an overview of transcriptome (**a**) and proteome (**b**) variability in different sample pairs. Transcripts and proteins with the most prominent changes in gene expression levels are located the furthest from zero on the y-axis. The heat maps show a global overview of changes in RNA levels of differentially expressed genes (**c**) and differentially translated proteins (**d**) at single gene/protein resolution. The gene/protein ranking is based on relative abundance detected at T0, with the most abundant transcripts appearing at the top of the heat map (red) and those with the lowest abundance appearing at the bottom (green). Differences in the expression/translation are given as Z-score values. Genes which were not expressed at a particular time point are presented in grey.

Figure 5 shows these molecular changes in *F. hepatica* eggs in a graphical form. Box plots (Fig. 5a,b) provide a summarised overview of the dynamics of the transcriptome and proteome at different points of development, where the height of the boxes represents an overall variation in RNA or protein levels. Figure 5a shows a low interquartile range (log_2_FC < 2.4), which indicates that 50% of significantly differentially expressed transcripts show minor changes in expression throughout the process of egg maturation. Only a few transcripts show major changes. Compared to the data from the other time points, T5–T10 show slightly less pronounced changes in transcript levels.

The same analysis was conducted for the proteome (Fig. 5b). As with the transcriptome, analysis of the proteomic data revealed that the number of changes in protein expression slightly decreases as time progresses, which is most noticeable when comparing changes between T0 and T10. Interestingly, the dispersion of upregulated and downregulated genes was much more symmetric in the transcriptomic data, while the proteome showed a clear tendency towards a higher number of downregulated proteins. For a full description (numerical representation of the box plots), see Supplementary file 6.

Heat maps (Fig. 5c,d) show an overall profile of these changes at individual protein-coding gene/protein resolution. Most striking changes in gene expression are found in a comparison between the T0 and T5 group. The variability of proteome (in terms of translated proteins) increased with age up to time point T5, while in 10-days-old eggs we did not detect a large part of proteins translated at earlier time points.

### Quantification of differentially expanded cysteine peptidases

**Figure 6:**
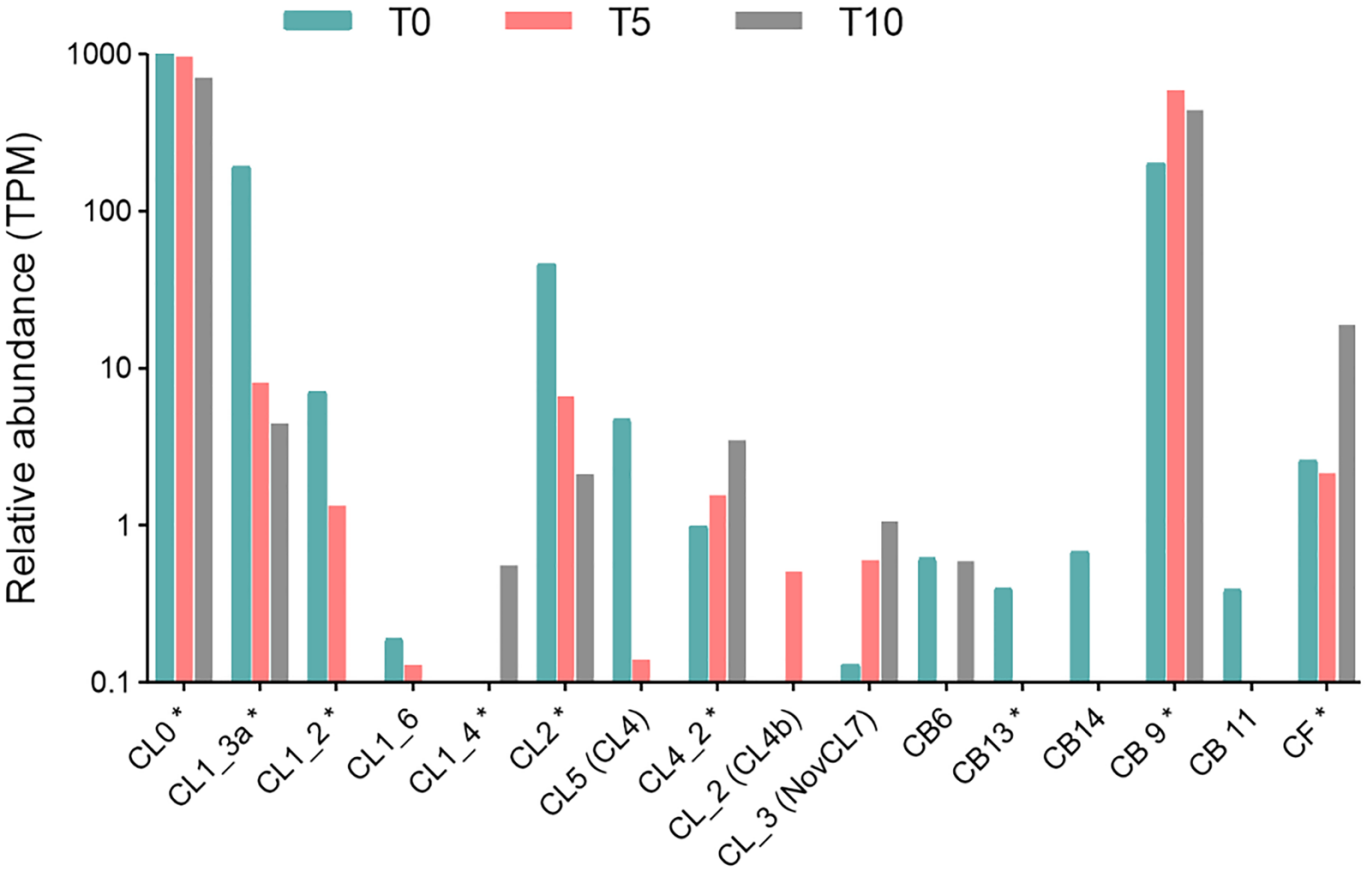
Relative expression of all transcripts coding cysteine cathepsins. Relative abundances (in TPM) are shown on a logarithmic scale. Isoforms marked by an asterisk were differentially expressed. Isoform CL1_3b was quantified only on protein level by LC–MS/MS. We used the Cwiklinski et al.^50^ nomenclature. Alternative cathepsins’ names used in McNulty et al.^13^ phylograms are shown in parentheses.

We performed a thorough exploration of cysteine cathepsin isoforms identified in the egg transcriptome/proteome. This was preceded by a manual curation of *F. hepatica* genome assembly (PRJEB25283) using previously described sequences^12,13,50^. In total, we identified 17 cysteine cathepsin isoforms, 16 of which were detected in the RNA-seq data. Two cathepsin L isoforms (CL0 and CL1_3a) and one cathepsin B isoform (CB9) belonged to the most expressed peptidase transcripts (Table 1). RNA-seq identified ten transcribed cathepsin L isoforms, five cathepsin B isoforms, and one cathepsin F isoform across all three developmental stages of the eggs (Fig. 6). A translation of five cathepsin L members was detected by LC–MS/MS (named CL0, CL2, CL1_3a, CL1_3b, and CL1_2; see Fig. 3), whereby the first four belonged to the ten most abundantly expressed peptidases (Table 1). Isoform CL1_3b was quantified only in LC–MS/MS data and it was the only differentially expressed cathepsin downregulated at T5 time point (Fig. 8).

### Differential expression analyses focused on peptidases

**Figure 7:**
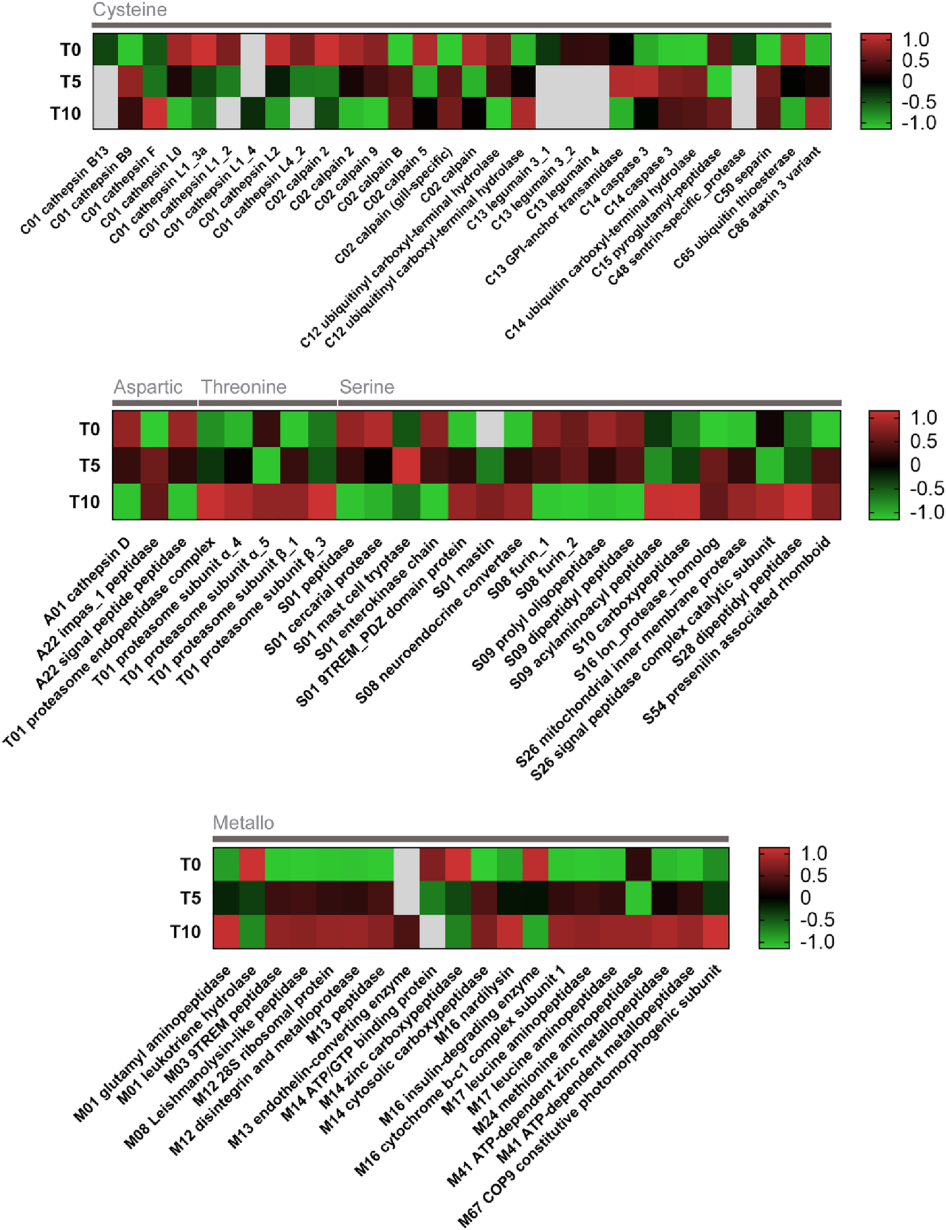
Expression dynamics of genes coding peptidases in *F. hepatica* eggs at different points of development. The heat maps show statistically significant changes in RNA levels of differentially expressed genes at a single gene resolution. Genes which are not expressed at a particular time point are coloured in grey. Differences in expression levels are shown as Z-score values. Gene names shown at x-axis begin with the peptidase family code; catalytic classes of peptidases are shown above the heatmap.

**Figure 8:**
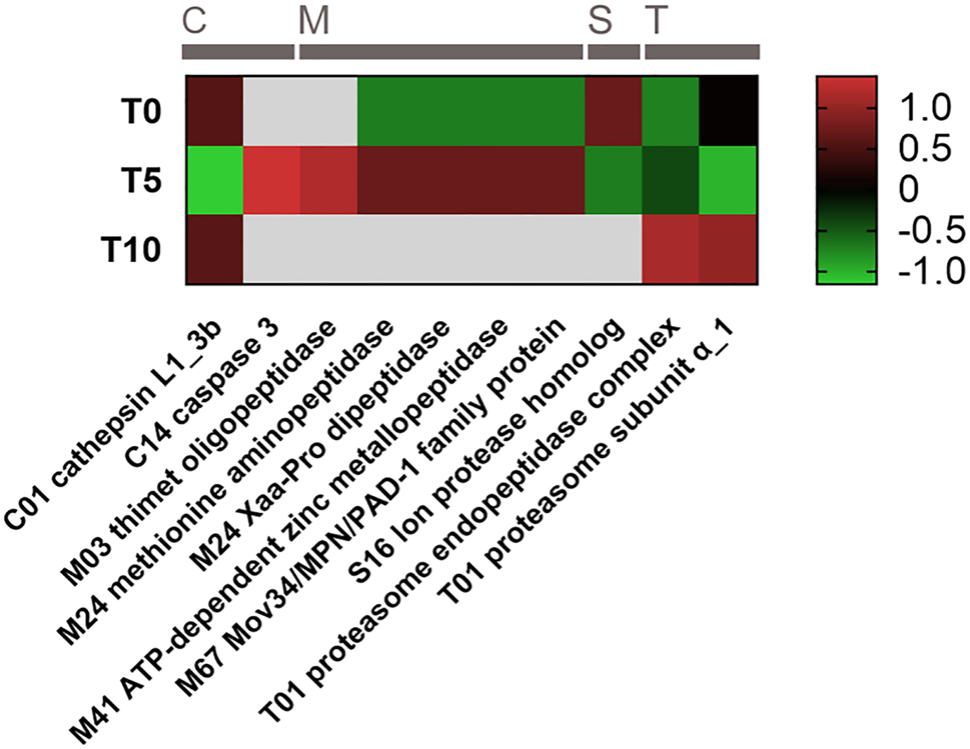
Statistically significant changes in the abundance of proteins with proteolytic activity based on LC–MS/MS quantification. The heat map shows statistically significant changes in protein abundance at a single gene resolution. Genes which are not expressed at a particular time point are coloured in grey. Differences in protein expression are shown as Z-score values. Gene names shown at x-axis begin with the peptidase family code; catalytic classes are shown above the heatmap (C—cysteine peptidases, M—metallopeptidases, S—serine peptidases, T—threonine peptidases).

Differential expression and translation of peptidases throughout the egg development was evaluated using the same approach as in the analyses of transcriptome and proteome (see above). The results are depicted in heatmaps (Figs. 7 and 8). Most cysteine peptidases were expressed in the freshly laid eggs (T0) (Fig. 7) but several cysteine peptidases (n = 12) were upregulated at later developmental stages, e.g., calpain B, separin, and ataxin (at stage T5) and cathepsin F (at T10). Nine cathepsin genes were found differentially expressed according to the RNA-seq data. Expression of cathepsins L isoforms decreases as the egg matures. After the initial upregulation of aspartic peptidase cathepsin D (one of the most expressed peptidases), its expression gradually decreased (from 3850 TPM at T0 to 2400 TPM at T10; Table 1). All isoforms of proteasome subunits formed by threonine peptidases were overexpressed at the T10 time point. Serine peptidases did not show a constant trend in terms of differential expression. For instance, furin, prolyl oligopeptidase, and dipeptidyl peptidase were expressed mainly directly after laying, while serine carboxypeptidase and lon protease homolog were upregulated in more mature eggs. Finally, metallopeptidases were overexpressed mainly in the T10 group, except for leukotriene hydrolase and zinc carboxypeptidase, which were upregulated at the time point T0. As for mass spectrometry data, only ten peptidases (or their isoforms) were found differentially translated (Fig. 8). Cathepsin L1_3a, upregulated in eggs soon after laying, was downregulated at T5, and then strongly expressed at T10 (for relative abundances, see Fig. 6). An overexpression of proteasome subunits was observed also in older eggs. Curiously, most of the differentially translated metallopeptidases were not detected in more mature eggs, but the trend of elevated production of metallopeptidases could be observed in 5-days-old eggs.

### Biochemical characterisation of proteolytic activities, pH optima, and inhibition assays

**Figure 9:**
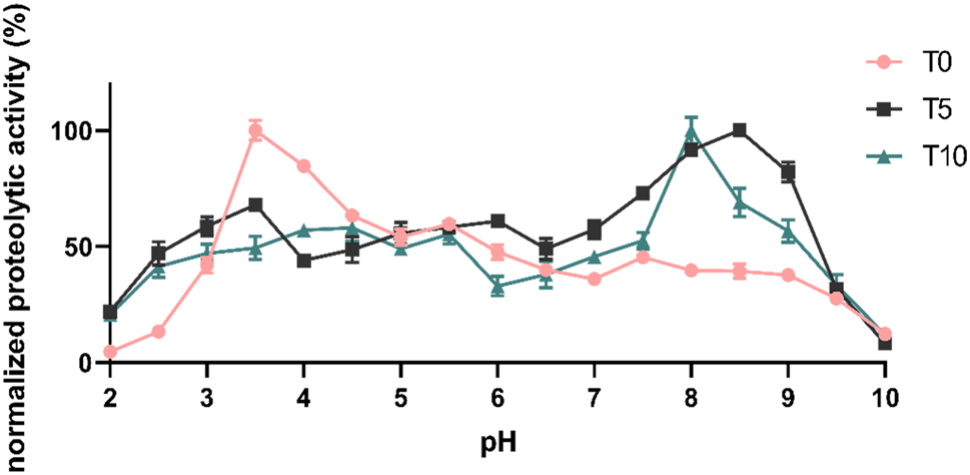
The pH profiles of proteolytic activity in extracts from *F. hepatica* eggs. Activities were measured using a library of FRET substrates in extracts from eggs collected 0, 5, and 10 days after laying (T0, T5, and T10, respectively). Data were normalised against the highest proteolytic activity recorded for each protein sample. Mean values are listed with the standard deviation.

Proteolytic activities in protein extracts from *F. hepatica* eggs were investigated also using a biochemical approach. Enzymatic activities were measured using a continuous kinetic assay with a complex library of internally quenched FRET substrates in buffers of a wide pH range (2–10). Proteolytic activities of extracts derived from eggs at different times after laying have different pH profiles (Fig. 9). The highest proteolytic activity of T0 eggs was detected in acidic conditions with a sharp optimum at pH 3.5 and two local optima at pH 5.5 and 7.5. At T5, on the other hand, the greatest activity was observed within pH range 8.0–9.0, and for T10 in alkaline pH around 8.0. Local optima were identified at pH 3.5 and 6.0 for T5 egg extract and around pH 4.5 and 5.5 for T10 egg extract. These findings indicate changes in the composition of peptidases during egg maturation.

**Figure 10:**
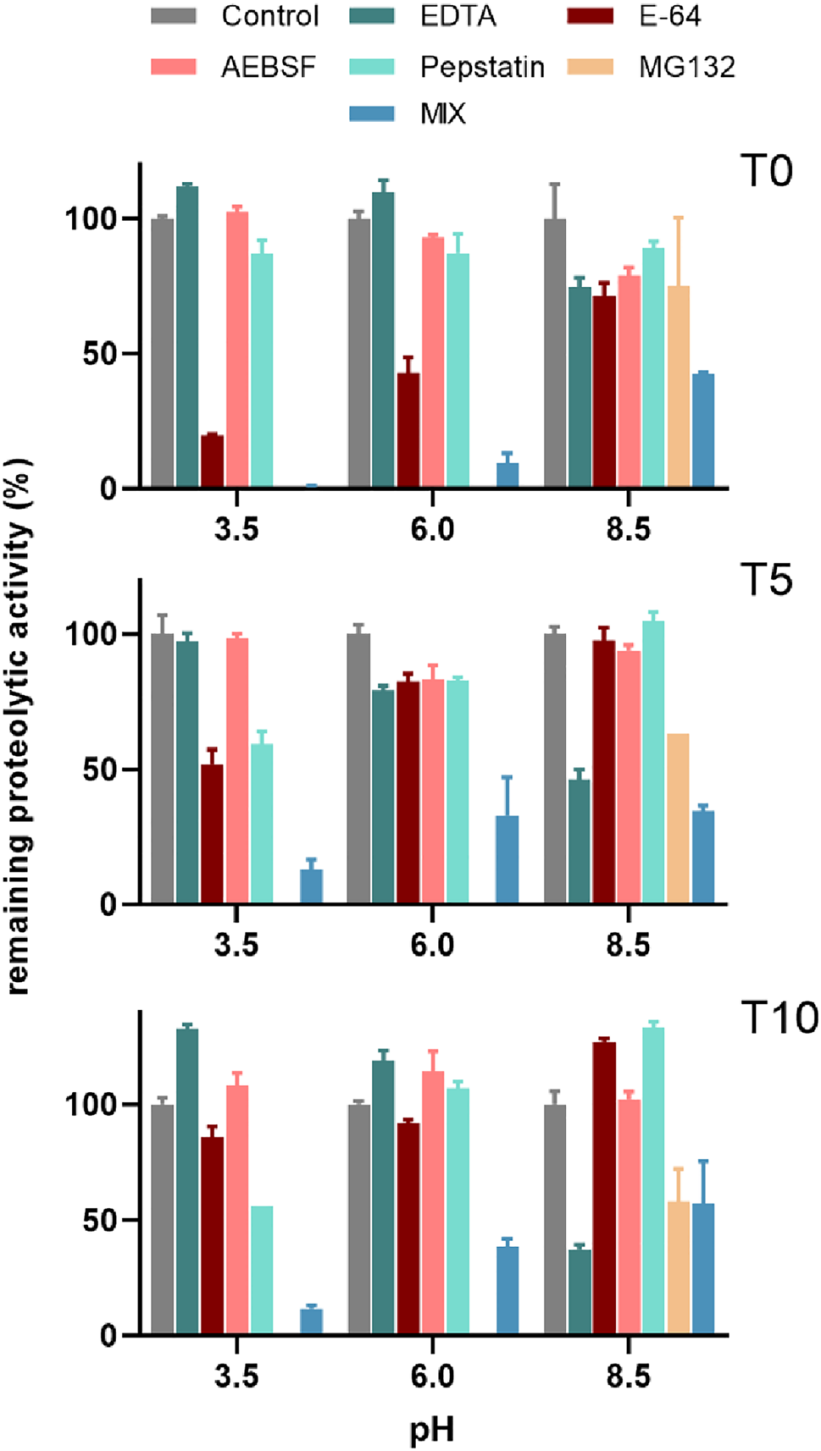
Inhibition of proteolytic activity in *F. hepatica* egg extracts using class-specific inhibitors. Proteolytic activity of protein extracts from *F. hepatica* eggs at 0, 5 and 10 days post laying (T0, T5 and T10, respectively) was assayed in the presence of class-specific inhibitors (EDTA, E-64, AEBSF, pepstatin, MG132) and a combination of all five inhibitors (MIX). Inhibitor of threonine peptidases (MG132) was used only at pH 8.5 due to its non-specific activity against cysteine peptidases in acidic environments. Inhibition of activity is expressed as the percentage of remaining proteolytic activity relative to a non-inhibited control (100%). Mean values are listed with standard deviation.

In order to identify which peptidase classes contribute to the global proteolytic activity in *F. hepatica* eggs, the protein extracts were treated with five class-specific peptidase inhibitors which selectively target metallopeptidases (EDTA)^54^, cysteine (l-trans-epoxysuccinyl-leucylamido(4-guanidino)butane, E-64)^55^, serine (4-(2-aminoethyl)benzenesulfonyl fluoride, AEBSF)^56^, aspartic peptidases (pepstatin)^57^, and threonine catalytic subunits of proteasome (benzyloxycarbonyl-l-leucyl-l-leucyl-leucinal, MG132)^58^. Reactions were performed at three different pH levels (3.5, 6, 8.5), which were selected according to the previously established pH optima of proteolytic activities of the egg extracts. Data are visualised in Fig. 10.

Cysteine peptidase inhibitor E-64 suppressed proteolysis at T0 in acidic pH 3.5 by 80%. A treatment of T5 and T10 samples with E-64 reduced the substrate turnover at this pH by 48% and 15%, respectively. It seems, therefore, that during the process of egg maturation the activity of cysteine peptidases gradually decreases. On the other hand, pepstatin, which inhibits aspartic peptidases, reduced the cleavage of substrate by 13%, 41%, and 44% at T0, T5, and T10, respectively, which indicates that egg development is accompanied by a growing presence of aspartic peptidases.

At mildly acidic pH 6, treatment with E-64 resulted in a 57% decrease of proteolytic activity in the T0 extract, which suggests a dominant role of cysteine peptidases. Inhibition by aspartic peptidase inhibitor pepstatin and serine peptidase inhibitor AEBSF caused only a slight decline in enzymatic activity (by 7% and 13%, respectively). Treatment of T5 extract with each of the five peptidase class inhibitors used in our study suppressed substrate cleavage by approx. 15–20%, while a combination of inhibitors reduced activity by approx. 65%, suggesting a complex contribution of different peptidase classes at T5. Analogous situation was observed in the T10 sample, where combined inhibitors of all five catalytic classes resulted in a 60% decrease in proteolytic activity.

Activity of metallopeptidases in extracts from eggs collected at all three time points was the strongest in alkaline environment (pH 8.5). EDTA provided increased inhibition by 25%, 54%, and 63% at T0, T5, and T10, respectively, which indicates a growing role of metallopeptidases during the process of egg development (similar to what was observed for aspartic peptidases at pH 3.5). A lower level of inhibition of activity by serine peptidase inhibitor AEBSF was observed in alkaline conditions in the T0 sample and in the T5 sample in neutral environment (by 20% and 16%, respectively), suggesting a role of serine peptidases. Moreover, alkaline pH creates optimal conditions for the activity of proteasome, whose subunits were identified among the most abundant peptidase-coding transcripts and translated peptidases (Table 1). Inhibition of activity at pH 8.5 with threonine peptidase inhibitor MG132 targeting proteasome resulted in a decreased activity by 25%, 40%, and 50% at T0, T5, and T10, respectively, which indicates that this group of peptidases is prevalent during the process of egg maturation. Incomplete inhibition in pH 6 and 8.5 indicates the presence of peptidases insensitive to the general class-specific inhibitors used in this study.

To conclude, our results showed that cysteine peptidases, which are active in acidic environments and prevalent especially in freshly laid eggs, are later partly replaced by aspartic peptidases. Egg maturation is accompanied by the initiation of metallopeptidase activity, which achieves its optimum in neutral and mildly alkaline environments. Threonine catalytic subunits of proteasome are active throughout the eggs’ development.

## Discussion

The eggs of fasciolids are well adapted to both the host and the outer environment, that is, two sets of conditions which vary in pH, temperature, biochemical composition, and in other respects. To respond effectively to these challenging, often unfavourable conditions, developmental stages of these parasites regulate the transcription of many genes. Unlike other intra-mammalian life-cycle stages, which were scrutinised using advanced high throughput sequencing technologies^11^, the embryonic stages of *F. hepatica* have not yet attracted such extensive research interest. To further our understanding of this developmental stage, we performed a comparative RNA-seq analysis aimed at identification of differentially expressed protein-coding transcripts, with a focus on peptidases, in eggs belonging to three stages: freshly laid eggs and eggs incubated for 5 and 10 days in 37 °C. This cultivation temperature was selected with the aim to mimic the environment of the host in order to monitor the molecular changes that take place during retention in the gall bladder and/or during the passage of eggs through the definitive host to the exterior environment. As shown by the viability assay, the exposure of freshly laid *F. hepatica* eggs to 37 °C for 5 and 10 days reduced their viability and ability to hatch. About 15% of eggs kept at 37 °C for 10 days were able to develop completely and release swimming miracidia, which indicates that the eggs retained within the host for days may continue to develop normally after being released to the external environment. Moreover, another 43% of these eggs have gone as far as the “eye spot” stage with moving embryos (25%) and cell division embryonating stage (18%). It is possible that the embryonation starts already within the host as evidenced by the eggs undergoing cell division observed after 5 days of incubation at 37 °C. It should be noted that other factors, such as pH, redox environment, presence of host enzymes, other molecules and elements which vary in bile ducts, gall bladder and different parts of intestine might contribute to the overall survival and development of the eggs. Hatchability of the control *F. hepatica* egg group (78%) is comparable with earlier studies conducted at temperature range 22–26 °C^34,59,60^.

Our study found a lower number of protein transcripts (7475, including 145 peptidases) than a study conducted on eggs from *F. hepatica* Uruguay isolate^13^ (9232 protein-coding transcripts, including 216 peptidases). In contrast to findings reported by Moxon et al.^21^, we did not observe a gradual increase in the variety of proteins with progressing egg development. In fact, the number of translated proteins quantified by LC–MS/MS was highest in 5-days-old eggs, and then it slightly declined. Our transcriptomic data do not support the raising molecular complexity either (in our study, 7005 transcripts were found at T0 vs. 6740 at T10).

In total, we identified 907 proteins, which is comparable with the 862 proteins observed in protein extracts from eggs of *Schistosoma japonicum*^61^. A majority of highly abundant proteins (43 out of 50, including alpha crystallin, GST, actin, thioredoxin peroxidase, or ferritin) was constitutively translated. This is in line with an earlier observation of stable production of 50 of the most abundant proteins^21^, which indicates that the main cellular components are constitutively expressed throughout egg development. Ten proteins reported by Moxon et al.^21^ were found among the first hundred most abundant proteins (highlighted in Supplementary file 5).

Proteins downregulated in aging eggs included for instance tubulin and fructose bisphosphate aldolase, while those intensely translated in 10 days old eggs were saposin-like protein, histone H4, or disulphide isomerase.

By combining transcriptomic and proteomic data, we aimed at a more precise analysis and functional annotation. Ultimately though, our data had shown that the dynamics of proteome does not always correspond to transcriptomic changes. A growing amount of evidence suggests that RNA abundances do not necessarily correlate with protein abundances^62,63^. It is possible that *F. hepatica* eggs can choose among several metabolic pathways to ensure optimal growth depending on current environmental conditions, which do often change. Changes in the transcription profile were most striking during the first 5 days of egg development.

Peptidases have different functions in a myriad of biological processes. According to the amino acid residue present in the active site of the peptidase, these enzymes can be grouped into seven catalytic types^64^. Previous studies have shown that serine, cysteine, aspartyl, and metallopeptidases from schistosome eggs participate in processes that result in excretion of the eggs from the host aided by the immune system response^52^. Of special importance in the case of trematodes are, due to their unique substrate specificity, cysteine cathepsins. *Fasciola* expanded the two types of cathepsin (L and B) into multigene families via gene multiplication^65^. The discrepancy between the number of peptidases predicted in our study (193) and that which was reported for *F. hepatica* Uruguay isolate^13^ (339) might be due to differences in the annotation methodology: while the abovementioned study annotated the dataset against the MEROPS database without any further filtering, we annotated the selected transcripts which were assigned GO terms related to ‘proteolysis’. This resulted in an exclusion of misleading annotations and a more accurate peptidase identification. Although earlier studies noted that peptidases are not importantly represented in *F. hepatica* eggs^21^, we detected active transcription of 145 peptidases in at least one studied time point. In our study, the predicted and quantified peptidase-coding transcripts represented about 2% of all protein-coding transcripts. These data are comparable with the study on the Uruguay isolate, where the predicted and quantified peptidase-coding transcripts were represented by 2.3% of all protein coding transcripts^13^. On the protein level, we were able to quantify 35 peptidases using LC–MS/MS, that is, 3.9% of all quantified proteins. Although we observed a relatively high variety of transcribed/translated genes coding peptidases, they represented between 10,000 to 13,000 TPM/PPM, respectively, that is about 1% and 1.3% of all quantified transcripts/proteins. This ratio is relatively low comparing to metacercariae and NEJ lifecycle stages, where transcripts encoding just cathepsins B1, B3, and legumain were represented by over 10,000 TPM^50^. The proportion of peptidases in terms of the sum of TPM/PPM values slightly increased in the course of the incubation process. Similarly, proteins associated with proteolytic activities were identified as overexpressed in mature eggs of *S. japonicum*^61^.

A more thorough exploration of egg peptidase composition had shown that cysteine peptidases were responsible for majority of proteolytic activity in acidic environment. While this activity diminished in the course of egg development, activity of aspartic peptidases, metallopeptidase, and proteasome increased. These changes were partially reflected in the transcriptomic data. Most genes coding for cysteine peptidases were downregulated as the eggs developed, while proteasome subunits and metallopeptidases were upregulated in older eggs. The most abundant peptidases detected in our study play a role in intracellular degradation of both lysosomal (cysteine peptidases) and cytoplasmic (threonine peptidases of proteasome) proteins.

Cysteine cathepsins of fasciolids, schistosomes, and opisthorchiids had undergone a remarkable amplification and diversification. They are vital for *F. hepatica* excystment, digestion, immune evasion, modulation, and migration. The varied proteolytic activity of these gene families reflects the changing requirements of lifecycle stages^65^ and their gene expression is meticulously regulated throughout the parasites’ lifecycle. For instance, stage-specific sequencing revealed that cathepsin B is most significantly overexpressed in metacercariae (cathepsin B), whereas isoforms of cathepsin L prevail in the NEJ. Both are secreted to the outer environment of the parasite, both are represented by over 10,000 TPMs^50^, and belong to the top five genes most significantly overexpressed by metacercariae. But existing knowledge of functions of these enzymes in both intramoluscan stages and parasite eggs is rather limited. Cwiklinski et al.^50^ clarified the number of known cathepsins by integrating the results of a number of previous studies^12–14^. Based on current genome assemblies, they identified 23 cathepsin L variants and 11 cathepsin B sequences, and corroborated them by RNA-seq^50^.

We examined the datasets in order to assess the variety of cysteine cathepsins expressed in developing eggs. Cathepsins L form five distinct clades (CL1–CL5) differring in residues in their S2 subsite which determine their substrate specificity. Additionally, a novel cathepsin L isoform (NovCL0) transcribed in eggs was reported in the *F. hepatica* Uruguay strain^13^. This cysteine peptidase is closely related to its vertebrate homolog, which was also the most abundant cathepsin L isoform detected in our samples: it is probably basal to all lineage-specific expansion. It was constitutively expressed with TPM reaching just under 1000 and relative abundance on the protein level at approx. 250 PPM. Additionally, RNA-seq confirmed the transcription of another nine cathepsin L transcripts. Three cathepsin L1 members and cathepsin L2 were also detected by LC–MS/MS, which supports the notion that the greatest expansion occurred among CL1 genes^14^. Cathepsin B9, the most basal of all cathepsins B^50^, which is also overexpressed in metacercariae, was relatively abundant in all three studied egg groups. Based on phylogenetic analyses, it used to be thought that *Fasciola* sp. produces only two types of clan C1 papain-like cysteine endopeptidases, namely cathepsin L and cathepsin B^66^, but a study by McNulty et al.^13^ reported also the existence of cathepsin F. Transcripts of this gene, which is highly amplified in opisthorchiids, were quantified also in our study (between 2–18 TPM).

Another important group of cysteine peptidases of trematodes are legumains (clan CD, family C13 asparaginyl endopeptidases), which are responsible for activation of cysteine cathepsins and highly abundant in the NEJ. Whereas *F. hepatica* genome assemblies revealed ten members of this gene family^12^, around one hundred isoforms have been estimated for *Opisthorchis viverrini*. In our study, minimal levels of legumain transcription in freshly laid eggs and the failure to detect them on the protein level at all indicate that *F. hepatica* eggs rely on other ways of cathepsin activation, such as autocatalytic cleavage at reduced pH.

Aspartic peptidase cathepsin D was intensely transcribed and translated in eggs of all studied time points in our study, but was not expressed in eggs from *F. hepatica* Uruguay isolate^13^. This endopeptidase, most active at acidic pH, contributes to lysosomal proteolysis and to the processing of antigens for the MHC class II system^67^.

Proteolytic activity in alkaline conditions was attributed mainly to threonine peptidase (proteasome) and metallopeptidases and was most prominent in eggs 10 days after laying. This trend was clearly observable in transcriptomic data but not recorded by proteomics. The relative abundance of members of the metallopeptidase family—such as leucine aminopeptidase (an immunodominant antigen known from adult *F. hepatica*^68^), pitrilysin, cytochrome b-c1 complex subunit with peptidase activity, and methionine aminopeptidase (with a role in cotranslational removal of N-terminal methionine in protein synthesis)—seldom reached 100 PPM. Expression of thimet oligopeptidase was recorded only in 5-days-old eggs. It is implicated in degrading peptides released by proteasomes and is also a significant factor limiting the extent of antigen presentation. Leucine aminopeptidase and thimet oligopeptidase were detected also in *S. mansoni* secretomes^33^, whereby, as shown by RNA interference, leucine aminopeptidase plays a crucial role in egg hatching process^69^.

Low protein expression and minimal proteolytic activity was observed in serine peptidases. Interestingly, three serine peptidases detected up to the fifth day post laying were missing completely in 10 day old eggs. Signalase, which cleaves signal peptides from selected proteins, was the most abundant serine peptidase. It should be noted that lon protease, which is able to cleave peptides resistant against enzymatic degradation^70^, was also expressed.

Proteins which play an important role in the host-parasite interaction often appear in the parasites’ excretory-secretory products (ESPs). In fact, catalytic activity is among the most highly enriched annotations of secretomes of schistosomes^33^ and peptidases are the most abundant class of proteins in the secretome of intra-mammalian stages of *F. hepatica*^50^. It would be interesting to investigate the *F. hepatica* egg secretome, but small size of these early developmental stages makes analysis of ESPs rather challenging. Our attempts to harvest sufficient amounts of ESPs for subsequent LC–MS/MS analysis have not been successful so far.

The function of most peptidases identified in helminth eggs remains enigmatic and this applies even to medically important parasites such as schistosomes or opisthorchids. In the present study, we combined a transcriptomic, proteomic, and biochemical approach to characterise changes in the proteolytic composition of *Fasciola hepatica* eggs during their development in the host. This report presents novel findings regarding changes in the transcriptome compared with the proteome during the different phases of *F. hepatica* egg development. Presented data can serve as a resource for further exploration of biology of this parasite which has a large impact on the wellbeing of both livestock and humans.

## Supplementary Information

Supplementary Information 1.

Supplementary Information 2.

Supplementary Information 3.

Supplementary Information 4.

Supplementary Information 5.

Supplementary Information 6.

## Data Availability

All data generated and analysed during this study are included in this article and its Supplementary Information files. Transcriptomic data discussed in this publication have been deposited in NCBI’s Gene Expression Omnibus^71^ and are accessible through GEO Series accession number GSE160622. Mass spectrometry proteomics data were deposited to the ProteomeXchange Consortium via PRIDE^72^ partner repository under dataset identifier PXD022516.
