# Supplementary material for: Transcriptomic and proteomic profiling of peptidase expression in Fasciola hepatica eggs developing at host’s body temperature
Source: Sci Rep. 2022 Jun 20;12:10308. doi: 10.1038/s41598-022-14419-z (PMC9209485; doi:10.1038/s41598-022-14419-z)
Supplement: Supplementary file 1 — Supplementary Information 1. [file 41598_2022_14419_MOESM1_ESM.docx]

**Supplementary file 1.** List of internally quenched substrates used to detect protease activity in protein homogenates from *F. hepatica* eggs.

| **Sequence^e^** | **Sequence^e^** |
| --- | --- |
| **1** | MCA-Pro-Pro-Gly-Phe-Ser-Ala-Phe-Thr-Lys(Dnp)-Gly-NH_2_^b^ |
| **2** | MCA-Tle-Gln-Ala-Ser-Ser-Arg-Ser-Lys(Dnp)-Gly-NH_2_ |
| **3** | MCA-Gly-Glu-Val-Ala-Arg-Pro-Leu-Gly-Lys(Dnp)-Gly-NH_2_ |
| **4** | MCA-Gly-Arg-Phe-Gly-Val-Trp-Lys-Ala-Lys(Dnp)-Gly-NH_2_ |
| **5** | MCA-Ser-Ala-Leu-Leu-Asn-Thr-Ser-Gly-Lys(Dnp)-Gly-NH_2_ |
| **6** | MCA-Ser-Glu-Val-Asn-Leu-Asp-Ala-Glu-Phe-Arg-Lys(Dnp)-Arg-Arg-NH_2_ |
| **7^b^** | MCA-Val-Asp-Val-Ala-Asp-Gly-Trp-Lys(DNP)-NH_2_ |
| **8^b^** | MCA-Tyr-Val-Ala-Asp-Ala-Pro-Lys(DNP)-NH_2_ |
| **9^c^** | MCA-Arg-Pro-Lys-Pro-Val-Glu-Nva-Trp-Arg-Lys(Dnp)-NH₂ |
| **10^d^** | MCA-Arg-Pro-Pro-Gly-Phe-Ser-Ala-Phe-Lys(Dnp)-OH |

^a^Substrates were synthesized as peptidyl amides by Fmoc solid-phase chemistry in an ABI 433A peptide synthesizer (Applied Biosystems). ^b, c, d^ Substrates were purchased from Sigma, Bachem, or R&D Systems, respectively. ^e^Abbreviations: MCA, 7-methoxycoumarin-4-yl acetic acid; Lys(Dnp), dinitro-phenyl lysine, Tle; L-tert-Leucine (L-α-t-butylglycine).
