# Supplementary material for: Transcriptomic and proteomic profiling of peptidase expression in Fasciola hepatica eggs developing at host’s body temperature
Source: Sci Rep. 2022 Jun 20;12:10308. doi: 10.1038/s41598-022-14419-z (PMC9209485; doi:10.1038/s41598-022-14419-z)
Supplement: Supplementary file 2 — Supplementary Information 2. [file 41598_2022_14419_MOESM2_ESM.docx]

**Supplementary file 2.** Illumina NextSeq 500 platform sequencing details.

| **Sample ID** | **Number of raw reads** | **Length (bp)** | **Length after trimming (bp)** | **Reads mapped exactly once (%)** | **Reads mapped more than once (%)** |
| --- | --- | --- | --- | --- | --- |
| T0, replicate 1 | 23,956,619 | 70 | 52 | 33.12 | 1.63 |
| T0, replicate 2 | 23,339,363 | 70 | 52 | 34.79 | 1.68 |
| T0, replicate 3 | 23,473,705 | 70 | 52 | 32.96 | 1.50 |
| T5, replicate 1 | 24,689,748 | 70 | 52 | 34.72 | 1.51 |
| T5, replicate 2 | 22,337,600 | 70 | 52 | 39.47 | 1.66 |
| T5, replicate 3 | 22,417,295 | 70 | 52 | 39.72 | 1.70 |
| T10, replicate 1 | 23,554,710 | 70 | 52 | 38.88 | 1.64 |
| T10, replicate 2 | 22,737,386 | 70 | 52 | 33.78 | 1.49 |
| T10, replicate 3 | 23,109,499 | 70 | 52 | 39.55 | 1.73 |
