# Supplementary material for: Transcriptomic and proteomic profiling of peptidase expression in Fasciola hepatica eggs developing at host’s body temperature
Source: Sci Rep. 2022 Jun 20;12:10308. doi: 10.1038/s41598-022-14419-z (PMC9209485; doi:10.1038/s41598-022-14419-z)
Supplement: Supplementary file 4 — Supplementary Information 4. [file 41598_2022_14419_MOESM4_ESM.docx]

| **GO term** | **Category** | **Function** |
| --- | --- | --- |
| GO:0003923 | Molecular Function | GPI-anchor transamidase activity |
| GO:0097263 | Molecular Function | Eoxin E4 synthase activity |
| GO:0036374 | Molecular Function | Glutathione hydrolase activity |
| GO:0002951 | Molecular Function | Leukotriene-C(4) hydrolase |
| GO:0019783 | Molecular Function | Ubiquitin-like protein-specific protease activity |
| GO:0019786 | Molecular Function | Atg8-specific protease activity |
| GO:0019785 | Molecular Function | ISG15-specific protease activity |
| GO:0019784 | Molecular Function | NEDD8-specific protease activity |
| GO:0016929 | Molecular Function | SUMO-specific protease activity |
| GO:0004843 | Molecular Function | Thiol-dependent ubiquitin-specific protease activity |
| GO:0004175 | Molecular Function | Endopeptidase activity |
| GO:0004176 | Molecular Function | ATP-dependent peptidase activity |
| GO:0004177 | Molecular Function | Aminopeptidase activity |
| GO:0004180 | Molecular Function | Carboxypeptidase activity |
| GO:0004181 | Molecular Function | Metallocarboxypeptidase activity |
| GO:0004185 | Molecular Function | Serine-type carboxypeptidase activity |
| GO:0004190 | Molecular Function | Aspartic-type endopeptidase activity |
| GO:0004197 | Molecular Function | Cysteine-type endopeptidase activity |
| GO:0004198 | Molecular Function | Calcium-dependent cysteine-type endopeptidase activity |
| GO:0004222 | Molecular Function | Metalloendopeptidase activity |
| GO:0004252 | Molecular Function | Serine-type endopeptidase activity |
| GO:0004298 | Molecular Function | Threonine-type endopeptidase activity |
| GO:0008233 | Molecular Function | Peptidase activity |
| GO:0008234 | Molecular Function | Cysteine-type peptidase activity |
| GO:0008235 | Molecular Function | Metalloexopeptidase activity |
| GO:0008236 | Molecular Function | Serine-type peptidase activity |
| GO:0008237 | Molecular Function | Metallopeptidase activity |
| GO:0008238 | Molecular Function | Exopeptidase activity |
| GO:0008239 | Molecular Function | Dipeptidyl-peptidase activity |
| GO:0008240 | Molecular Function | Tripeptidyl-peptidase activity |
| GO:0008241 | Molecular Function | Peptidyl-dipeptidase activity |
| GO:0008242 | Molecular Function | Omega peptidase activity |
| GO:0008798 | Molecular Function | Beta-aspartyl-peptidase activity |
| GO:0009002 | Molecular Function | Serine-type D-Ala-D-Ala carboxypeptidase activity |
| GO:0009046 | Molecular Function | Zinc D-Ala-D-Ala carboxypeptidase activity |
| GO:0016805 | Molecular Function | Dipeptidase activity |
| GO:0016807 | Molecular Function | Cysteine-type carboxypeptidase activity |

**Supplementary file 4.** List of selected GO terms related to the proteolytic activity.

| **GO term** | **Category** | **Function** |
| --- | --- | --- |
| GO:0016920 | Molecular Function | Pyroglutamyl-peptidase activity |
| GO:0034701 | Molecular Function | Tripeptidase activity |
| GO:0034722 | Molecular Function | Gamma-glutamyl-peptidase activity |
| GO:0042500 | Molecular Function | Aspartic endopeptidase activity, intramembrane cleaving |
| GO:0045148 | Molecular Function | Tripeptide aminopeptidase activity |
| GO:0061473 | Molecular Function | Murein tripeptide carboxypeptidase activity |
| GO:0061785 | Molecular Function | Peptidoglycan endopeptidase activity |
| GO:0061786 | Molecular Function | Peptidoglycan stem peptide endopeptidase activity |
| GO:0061787 | Molecular Function | Peptidoglycan cross-bridge peptide endopeptidase activity |
| GO:0070001 | Molecular Function | Aspartic-type peptidase activity |
| GO:0070002 | Molecular Function | Glutamic-type peptidase activity |
| GO:0070003 | Molecular Function | Threonine-type peptidase activity |
| GO:0070004 | Molecular Function | Cysteine-type exopeptidase activity |
| GO:0070005 | Molecular Function | Cysteine-type aminopeptidase activity |
| GO:0070006 | Molecular Function | Metalloaminopeptidase activity |
| GO:0070007 | Molecular Function | Glutamic-type endopeptidase activity |
| GO:0070008 | Molecular Function | Serine-type exopeptidase activity |
| GO:0070009 | Molecular Function | Serine-type aminopeptidase activity |
| GO:0070012 | Molecular Function | Oligopeptidase activity |
| GO:0070122 | Molecular Function | Isopeptidase activity |
| GO:0070137 | Molecular Function | Ubiquitin-like protein-specific endopeptidase activity |
| GO:0070138 | Molecular Function | Ubiquitin-like protein-specific isopeptidase activity |
| GO:0070139 | Molecular Function | SUMO-specific endopeptidase activity |
| GO:0070140 | Molecular Function | SUMO-specific isopeptidase activity |
| GO:0070573 | Molecular Function | Metallodipeptidase activity |
| GO:0071972 | Molecular Function | Peptidoglycan L,D-transpeptidase activity |
| GO:0097153 | Molecular Function | Cysteine-type endopeptidase activity involved in apoptotic process |
| GO:0097199 | Molecular Function | Cysteine-type endopeptidase activity involved in apoptotic signaling pathway |
| GO:0097200 | Molecular Function | Cysteine-type endopeptidase activity involved in execution phase of apoptosis |
| GO:0102008 | Molecular Function | Cytosolic dipeptidase activity |
| GO:0102009 | Molecular Function | Proline dipeptidase activity |
| GO:0102274 | Molecular Function | Glutathione S-conjugate carboxypeptidase activity |
| GO:0103046 | Molecular Function | Alanylglutamate dipeptidase activity |
| GO:1902944 | Molecular Function | Aspartic-type endopeptidase activity involved in amyloid precursor protein catabolic process |
| GO:1902945 | Molecular Function | Metalloendopeptidase activity involved in amyloid precursor protein catabolic process |
| GO:0006508 | Biological Process | Proteolysis |
|  |  |  |
