# Supplementary material for: Transcriptomic and proteomic profiling of peptidase expression in Fasciola hepatica eggs developing at host’s body temperature
Source: Sci Rep. 2022 Jun 20;12:10308. doi: 10.1038/s41598-022-14419-z (PMC9209485; doi:10.1038/s41598-022-14419-z)
Supplement: Supplementary file 6 — Supplementary Information 6. [file 41598_2022_14419_MOESM6_ESM.docx]

**Supplementary file 6.** Numeric representation of the box plots. Numbers are based on statistically significant changes (adjusted P-values cut-off 0.05) in the transcriptome and proteome variability in *F. hepatica* eggs of different ages.

|  | **Transcriptomics data** | | | **Proteomics data** | | |
| --- | --- | --- | --- | --- | --- | --- |
|  | **T0-T5** | **T0-T10** | **T5-T10** | **T0-T5** | **T0-T10** | **T5-T10** |
| **Sample size** | 1936 | 3329 | 611 | 75 | 18 | 244 |
| **Median** | 0.405 | -0.479 | -0.650 | -1.992 | -5.179 | -2.396 |
| **Mean** | 0.336 | 0.056 | -0.027 | -0.468 | -5.198 | -1.709 |
| **Minimum** | -3.645 | -4.765 | -2.941 | -5.800 | -7.318 | -5.855 |
| **Maximum** | 3.979 | 4.633 | 3.104 | 4.210 | -2.743 | 3.040 |
| **1. quartile** | -0.794 | -1.267 | -0.829 | -3.545 | -6.295 | -3.734 |
| **3. quartile** | 1.150 | 1.094 | 0.769 | 3.252 | -4.273 | 0.770 |
